# Bmi-1 promotes the aggressiveness of glioma via activating the NF-kappaB/MMP-9 signaling pathway

**DOI:** 10.1186/1471-2407-12-406

**Published:** 2012-09-11

**Authors:** Lili Jiang, Jueheng Wu, Yi Yang, Liping Liu, Libing Song, Jun Li, Mengfeng Li

**Affiliations:** 1 Department of Pathophysiology, Guangzhou Medical University, Guangzhou, Guangdong 510182 China; 2 Department of Microbiology, Zhongshan School of Medicine, Sun Yat-sen University, Guangzhou, Guangdong 510080, China; 3 Key Laboratory of Tropical Disease Control (Sun Yat-sen University), Chinese Ministry of Education, Guangzhou, Guangdong 510080, China; 4 Department of Pharmacology, Zhongshan School of Medicine, Sun Yat-sen University, Guangzhou, Guangdong 510080, China; 5 Department of Experimental Research, Cancer Center, Sun Yat-sen University, Guangzhou, Guangdong 510060, China; 6 Department of Biochemistry, Zhongshan School of Medicine, Sun Yat-sen University, Guangzhou, Guangdong 510080, China; 7 Zhongshan School of Medicine, Sun Yat-sen University, 74 Zhongshan Road II, Guangzhou, Guangdong 510080, China

**Keywords:** Bmi-1, Glioma, Invasion, MMP-9, NF-kappaB

## Abstract

**Background:**

The prognosis of human glioma is poor, and the highly invasive nature of the disease represents a major impediment to current therapeutic modalities. The oncoprotein B-cell-specific Moloney murine leukemia virus integration site 1 protein (Bmi-1) has been linked to the development and progression of glioma; however, the biological role of Bmi-1 in the invasion of glioma remains unclear.

**Methods:**

A172 and LN229 glioma cells were engineered to overexpress Bmi-1 via stable transfection or to be silenced for Bmi-1 expression using RNA interfering method. Migration and invasiveness of the engineered cells were assessed using wound healing assay, Transwell migration assay, Transwell matrix penetration assay and 3-D spheroid invasion assay. MMP-9 expression and activity were measured using real-time PCR, ELISA and the gelatin zymography methods. Expression of NF-kappaB target genes was quantified using real-time PCR. NF-kappaB transcriptional activity was assessed using an NF-kappaB luciferase reporter system. Expression of Bmi-1 and MMP-9 in clinical specimens was analyzed using immunohistochemical assay.

**Results:**

Ectopic overexpression of Bmi-1 dramatically increased, whereas knockdown of endogenous Bmi-1 reduced, the invasiveness and migration of glioma cells. NF-kappaB transcriptional activity and MMP-9 expression and activity were significantly increased in Bmi-1-overexpressing but reduced in Bmi-1-silenced cells. The reporter luciferase activity driven by *MMP-9* promoter in Bmi-1-overexpressing cells was dependent on the presence of a functional NF-kappaB binding site, and blockade of NF-kappaB signaling inhibited the upregulation of MMP-9 in Bmi-1 overexpressing cells. Furthermore, expression of Bmi-1 correlated with NF-kappaB nuclear translocation as well as MMP-9 expression in clinical glioma samples.

**Conclusions:**

Bmi-1 may play an important role in the development of aggressive phenotype of glioma via activating the NF-kappaB/MMP-9 pathway and therefore might represent a novel therapeutic target for glioma.

## Background

Glioma is a common type of primary brain tumor and represents one of the most aggressive and lethal human cancer types [1]. Despite of enormous advances in surgical techniques and development of therapeutic agents, the mortality of glioma remains high, with a cumulative 1-year survival rate lower than 30% [2,3]. The poor survival of glioma patients is largely attributed to the highly invasive phenotype of glioma cells, which has been associated with the widely recognized difficulty of performing complete surgical resection of gliomas [4]. At the molecular level, tumor cell invasion is mediated by sets of factors that initiate or promote cell motility, matrix destruction, angiogenesis and other biological events [5-8]. Matrix metalloproteinase-9 (MMP-9), a member of the matrix metalloproteinase class of gelatinases, plays essential roles in the invasiveness of glioma cells, mainly by catalyzing the destruction of basal membrane and extracellular matrix [9-11].

B-cell-specific Moloney murine leukemia virus integration site 1 protein (Bmi-1) acts as a repressor of the expression of certain genes by forming complexes with multiple other Polycomb group (PcG) family members, such as RING1, HPC2 and Mph2[12]. It has been reported that Bmi-1 represses cells senescence by acetylhydrolysis or deacetylhydrolysis of polycomb response elements in chromosomes [13,14]. Numerous experimental studies have indicated that Bmi-1 plays an important role in the development and progression of cancer and essentially functions as an oncogene [15,16]. Knocking down Bmi-1 induces cell-cycle arrest and rescues the mRNA levels of tumor-suppressive *p16*^*INK4a*^*homeobox A9* (*HOXA9*) and *homeobox C13* (*HOXC13*) genes [14,17]. In contrast, overexpression of Bmi-1 prevents cancer cell apoptosis, possibly by activation of nuclear factor kappaB (NF-kappaB) pathway signaling [18]. Moreover, various studies have revealed that Bmi-1 is required for the maintenance of the self-renewing proliferation of several normal and cancer stem cells, including neural crest stem cells and mammary stem cells [19,20].

Aberrant activation of NF-kappaB is observed in various types of human cancer, including glioma. Nuclear localization of p65, an indicator of NF-kappaB activation, has also been demonstrated in clinical specimens of glioblastoma multiform (GBM) [8,18,21]. The NF-kappaB signialing orchestrates several key biological processes during the development and progression of cancer by inducing transcription of a variety of target genes that regulate cell proliferation, survival, invasion and angiogenesis [8,18,22-25]. Inhibition of NF-kappaB activation enhances the radiosensitivity of human glioma cells, and also inhibits the proliferation and invasiveness of glioblastoma cells and glioblastoma-induced angiogenesis [26,27]. Nevertheless, whether, and how, the biological functions of NF-kappaB is involved in the oncogenic role of Bmi-1 remains obscure.

In the present study, we observed that overexpression of Bmi-1 promoted, whereas knockdown of Bmi-1 inhibited, the invasion and migration of glioma cells. We also demonstrated that the ability of Bmi-1 to stimulate the invasive phenotype in glioma cells was mechanistically associated with activation of NF-kappaB and subsequent upregulation and activation of MMP-9.

## Methods

### Cells and cell treatments

Glioma cell lines LN229 and A172 were grown in Dulbecco’s modified Eagle’s medium (Invitrogen, Carlsbad, CA) supplemented with 10% fetal bovine serum (FBS) (HyClone, Logan, UT), 100 units penicillin and 100 units streptomycin at 37°C in 5% CO_2_ atmosphere in a humidified incubator. The MMP inhibitor and NF-kappaB activation inhibitor II JSH-23 (EMD, La Jolla, CA) compounds were dissolved in dimethyl sulfoxide (DMSO) and used, respectively, at 50μM and 30μM. Treatment of cells with the MMP inhibitor was performed for indicated time lengths, and JSH-23 was used to treat cells for 11 h.

### Vectors and retroviral infection

pMSCV/Bmi-1 overexpressing human Bmi-1 was constructed as previously described [15]. To silence endogenous Bmi-1 expression, Bmi-1 RNA interference (RNAi) sequence (5’-ATGAAGAGAAGAAGGGATT-3’, synthesized by Invitrogen) was cloned into retroviral transfer vector pSuper-retro-puro. As described previously [28], retroviral particles were produced by cotransfection with pSuper-retro-Bmi-1-shRNA and PIK packaging plasmid into 293 T cells and collected 24 to 48 hrs after transfection to infect glioma cells. Stable cell lines expressing Bmi-1 or with Bmi-1 silenced were selected by treatment with 0.5 μg/ml puromycin for 10 days, beginning from 48 hours after infection [28]. After selection for 10–14 days, the cell lysates prepared from the pooled population of cells in the sampling buffer were fractionated on SDS-PAGE for immunoblotting detection of Bmi-1 protein level.

### Real-time RT-PCR and data analysis

Total cellular RNA was extracted using the Trizol reagent (Invitrogen) according to the manufacturer’s instruction. Two micrograms of RNA from each sample were used for cDNA synthesis primed with random hexamers. For PCR amplification of cDNA, an initial amplification using gene-specific primers was done with a denaturation step at 95°C for 10 minutes, followed by 28 cycles of denaturation at 95°C for 60 seconds, primer annealing at 58°C for 30 seconds, and primer extension at 72°C for 30 seconds. At completion of the cycling, a final extension at 72°C for 5 minutes was done before the reaction was terminated. Expression levels of genes were normalized to housekeeping gene GAPDH as the control. PCR primers were designed by employing the Primer Express version 2.0 software (Applied Biosystems, Foster City, CA). The Primers was shown as following: *MMP9-up*: ACGACGTCTTCCAGTACCGA; *MMP9-dn*: TTGGTCCACCTGGTTCAACT; *CCND1-up*: AACTACCTGGACCGCTTCCT; *CCND1-dn*: CCACTTGAGCTTGTTC ACCA; *Bcl-xL-up*: ATTGGTGAGTCGGATCGCAGC; *Bcl-xL-dn*: AGAGAAGGGGG TGGGAGGGTA; *TNF alpha-up*: CCAGGCAGTCAGATCATCTTCTC; *TNF alpha-dn*: AGCTGGTTATCTCTCAGCTCCAC; *VEGFC-up*: GTGTCCAGTGTAGATGAACTC; *VEGFC-dn*: ATCTG TAGACGGACACACATG; *MYC-up:* TTCGGGTAGTGGAAAACCAG; *MYC-dn:* CAGCAGCTCGAATTTCTTCC; *GAPDH-up*: GACTCATGACCACAGTCCATGC; *GAPDH-dn*: AGAGGCAGGGATGATGTTCTG.

### Western blotting analysis

Western blotting analysis was performed according to standard methods as previously described [18].The membrane was probed with a 1:500-diluted rabbit anti-human Bmi-1 antibody (Cell Signaling, Danvers, MA). The membranes were stripped and re-probed with a mouse anti-β-actin monoclonal antibody (1:1,000; Sigma, Saint Louis, MI) as a loading control.

### Wound healing assay

Cells were seeded on six-well plates with DMEM containing 10% FBS and grown to confluence. The cells were scratched with a sterile 200μL pipette tip to create artificial wounds. At 0 and 24 hr after wounding, respectively, phase-contrast images of the wound healing process were photographed digitally using an inverted Olympus IX50 microscope with 10× objective lens. Eight images per treatment were analyzed to determine averaging parameters of positioning of the migrating cells at the wound edges by digitally drawing lines using the Image-Pro Plus software (Media Cybernetics).

### Transwell migration assay and Transwell matrix penetration assay

Cells (1 × 10^4^) to be tested were plated on the top side of the polycarbonate Transwell filter without (for Transwell migration assay) or with Matrigel coating (for Transwell matrix penetration assay) in the upper chamber of the BioCoat^TM^ Invasion Chambers (BD, Bedford, MA) and incubated at 37°C for 22 hrs, followed by removal of cells inside the upper chamber with cotton swabs. Migrated and invaded cells on the membrane bottom-surface were fixed in 1% paraformaldehyde, stained with hematoxylin, and counted (Ten random 200× fields per well). Cell counts were expressed as the mean number of cells per field of view. Three independent experiments were performed and the data are presented as mean ± standard deviation (SD).

### 3-D spheroid invasion assay

The Matrigel matrix (BD Biosciences, San Jose, CA) was used in 3-D spheroid invasion assay, which displays morphologies typical of highly aggressive invasiveness presenting more outward projections (Invadopodia or invasive feet) [29-33]. Indicated cells (1 × 10^4^) were trypsinized and seeded in 24-well plates coated with Matrigel (2%, BD Biosciences), and medium was changed every other day. Pictures were taken under microscope at 2-day intervals for 2–3 weeks.

### Immunohistochemical analysis (IHC)

IHC was performed according to standard methods as previously described [28]. Sections were IHC analyzed using anti-Bmi-1, anti-MMP-9 and anti-NF-kappaB antibodies (Cell signaling, Danvers, MA,). Images were captured using the AxioVision Rel.4.6 computerized image analysis system (Carl Zeiss Co. Ltd., Jena).

### Luciferase assay

Cells (1.5 × 10^4^) were seeded in triplicates in 24-well plates and allowed to settle for 24 hrs. One hundred nanograms of luciferase reporter plasmid containing fragments of the MMP-9 promoter with serial deletions, pNF-kappaB-luc plasmid, or the control-luciferase plasmid, in combination with 1 ng of pRL-TK renilla plasmid (Promega,Madison, WI), were transfected into glioma cells using the Lipofectamine 2000 reagent (Invitrogen, Co., Carlsbad, CA) according to a protocol provided by the manufacturer. Luciferase and renilla signals were measured at 48 h after transfection using the Dual Luciferase Reporter Assay Kit (Promega, Madison, WI) according to the manufacturer’s instruction. Three independent experiments were performed and the data are presented as mean ± SD.

### Enzyme-linked immunosorbent assay (ELISA)

ELISA was performed using a commercial kit according to the manufacturer’s manual (Keygentec, Shanghai). Briefly, 100 μl of diluted standard and tested samples, namely, Bmi-1-overexpressing or -silencing cells, and the vector control cells, including a negative control, were added to the ELISA plate and incubated at 36°C for 90 min. After the unbound samples were washed off by DI water and PBS-Triton, specific antibody (anti-MMP-9, anti-MMP-2, or anti-MMP-7) was incubated with the plate at 36°C for 60 min. After further washing steps, 100 μl of second antibody was added and incubated for 60 min. Subsequently, the substrate was added and incubated at RT for 60 min before the reaction was stopped, followed by the results-reading with a microplate reader. Colorimetric measurement was recorded as OD_450_ readings.

### Gelatin zymography assay

Cells were seeded in 48-well culture plates at a density of 3 × 10^4^/well and incubated for 24 h before the medium was replaced with serum-free medium (Invitrogen, Carlsbad, CA), followed by collection of conditioned medium and quantification for protein contents. Samples containing equal amounts of protein (1 μg/μl) mixed with 4 × sampling buffer (3:1) were run on 9% polyacrylamide gels containing 0.2% gelatin (Sigma, Saint Louis, MI). After electrophoresis, the gel was washed twice in wash buffer (2.5% Triton X-100, 50 mM Tris–HCl, 1 μM ZnCl_2_, pH 7.6) for 45 min/each time, followed by two rinses with the wash buffer (without Triton X-100) and subsequent incubation at 37°C in 50 mM Tris–HCl (pH7.6), 5 mM CaCl_2_, 1 μM ZnCl_2_ and 0.02% Brij-35 for 16 h. The gels were stained with 0.1% Coomassie brilliant blue R-250 and then de-stained with de-staining solution (40% methanol, 10% acetic acid in distilled water). A protein marker was used to measure the molecular weights of proteins in SDS-PAGE before the staining and washing step of the Zymography Assay. Meanwhile, the MMP-9 recombinant protein was also used as the standard to confirm the band of MMP-9.

### Tissue specimens and patient information

Paraffin-embedded, archived glioma specimens were histopathologically diagnosed at the First Affiliated Hospital of Sun Yat-sen University from 2000 to 2005. The clinical information is described in Additional file 1: Table S1. The use of the clinical specimens was approved by the local Institutional Review Board, the Ethical Committee of the First Affiliated Hospital of Sun Yat-sen University, Guangzhou, Guangdong, China, and conformed to the ethical guidelines of the Helsinki Declaration.

### Statistical analysis

Statistical analyses were performed using the SPSS 11.0 statistical software package. Data represent mean ± SEM. P values of 0.05 or less were considered statistically significant.

## Results

### Bmi-1 induced the aggressive phenotype in glioma cells *in vitro*

**Figure 1:**
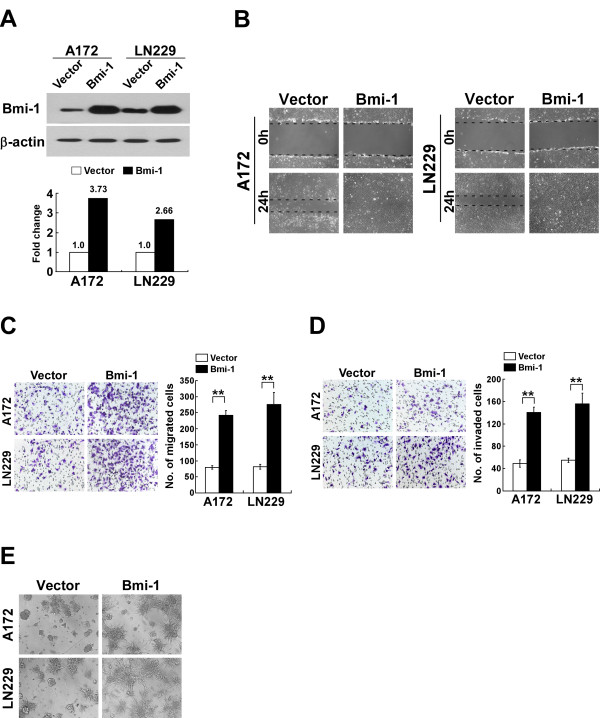
** Ectopic expression of Bmi-1 enhances the migration and invasion of glioma cells. A**, Western blot of Bmi-1 protein expression in A172-vector, A172-Bmi-1, LN229-vector and LN229-Bmi-1 cells; β-actin was used as a loading control (upper). The fold changes of Bmi-1 expression were analyzed by densitometry quantification (lower). **B**, Wound healing assay of A172-vector, A172-Bmi-1, LN229-vector and LN229-Bmi-1 cells. **C**, Representative micrographs (left) and quantification (right) of cell migration in the Transwell migration assay (without matrigel). **D**, Representative micrographs (left) and quantification (right) of cell invasion in the Transwell matrix penetration assay (with matrigel). **E**, Representative micrographs from the three-dimensional spheroid invasion assay on the 4^th^ day after cells were planted; these experiments were repeated at least three times with similar results. Vector: pMSCV-vector. Error bars represent the mean ± SD of three independent experiments; ** *P* < 0.01.

To investigate the effect of Bmi-1 on the aggressiveness of glioma cells, A172 and LN229 glioma cells stably overexpressing Bmi-1 were established (Figure 1A). Wound healing assays demonstrated that ectopic expression of Bmi-1 accelerated the migration of glioma cells (Figure 1B). Transwell migration assay (without Matrigel) revealed that overexpression of Bmi-1 significantly increased the rate of migration of A172 and LN229 glioma cells, as compared with that of control cells (Figure 1C). Furthermore, the Transwell matrix penetration assay (coated with Matrigel) showed that overexpression of Bmi-1 increased the invasive ability of both glioma cell lines (Figure 1D). Strikingly, the 3-D spheroid invasion assay demonstrated that Bmi-1-overexpressing glioma cells displayed cellular morphologies typical of a highly invasive phenotype, as the cells presented increased numbers of outward projections compared to the control cells (Figure 1E). Taken together, these data suggested that overexpression of Bmi-1 promoted the migration and invasiveness of glioma cells *in vitro*.

### Bmi-1 increased MMP-9 expression and activity in glioma cells

**Figure 2:**
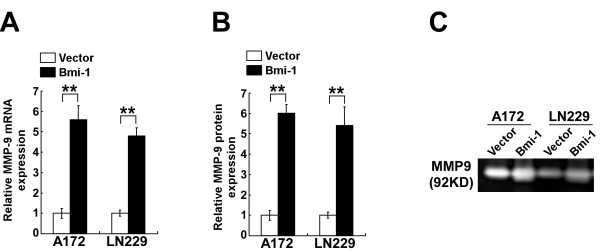
** Bmi-1 activates MMP-9 in glioma cells. A**, Real-time PCR quantification of *MMP-9* mRNA expression levels in vector-control cells and Bmi-1-overexpressing cells (Bmi-1). *MMP-9* expression levels are presented as the fold changes relative to that in vector-control cells and normalized to GAPDH. **B**, ELISA for secreted MMP-9 protein in cell supernatants. **C**, Gelatin zymography assay of MMP-9 gelatinase activity in cell supernatants. Vector: pMSCV-vector. Error bars represent the mean ± SD of three independent experiments; ** *P* < 0.01.

Numerous reports have mechanistically associated the invasive ability of glioma cells with expression and activation of MMP-9 [34]. To understand the mechanism by which overexpression of Bmi-1 promoted the invasiveness and migration of glioma cells, we investigated the expression and activity of MMP-9 in A172 and LN229 glioma cells stably overexpressing Bmi-1. As shown in Figure 2A*MMP-9* mRNA expression was upregulated in Bmi-1-overexpressing cells compared to that in control cells. ELISA (Figure 2B) and the gelatin zymography assay confirmed that overexpression of Bmi-1 increased MMP-9 expression and activity in glioma cells (Figure 2C). Taken together, these data suggested that overexpression of Bmi-1 upregulated and activated MMP-9 in glioma cells *in vitro*.

### Silencing Bmi-1 reduced glioma cell invasiveness and MMP-9 expression

**Figure 3:**
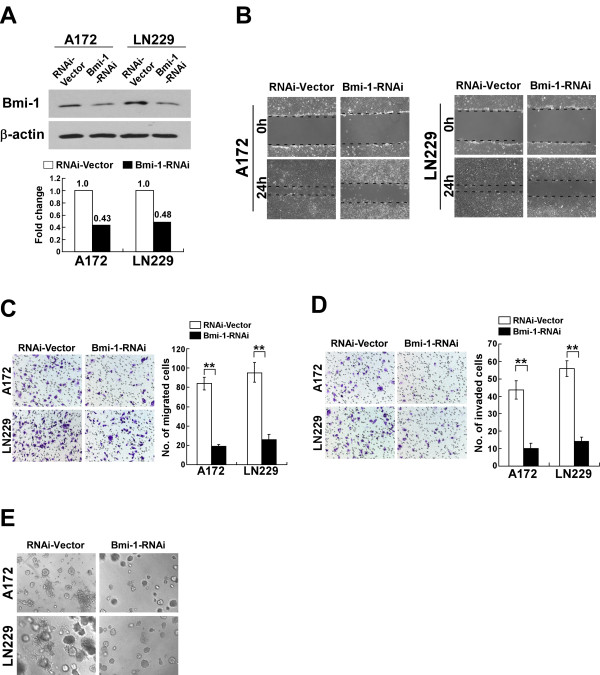
** Knockdown of Bmi-1 reduces the migration and invasion of glioma cells. A**, Western blot analysis of Bmi-1 protein expression in vector-control cells and Bmi-1-shRNA-transduced glioma cell lines (Bmi-1-RNAi); β-actin was used as a loading control (upper). The fold changes of Bmi-1 expression were analyzed by densitometry quantification (lower). **B**, Wound healing assay of vector-control cells and Bmi-1-shRNA-transduced glioma cell lines. **C**, Representative micrographs and quantification of cell migration in the Transwell migration assay (without matrigel). **D**, Representative micrographs and quantification of cell invasion in the Transwell matrix penetration assay (with matrigel). **E**, Representative micrographs from the three-dimensional spheroid invasion assay on the 4^th^ day after cells were planted. The experiments were repeated for at least three times with similar results. RNAi-Vector: pSuper-retro-puro-vector. Error bars represent the mean ± SD of three independent experiments; ** *P* < 0.01.

**Figure 4:**
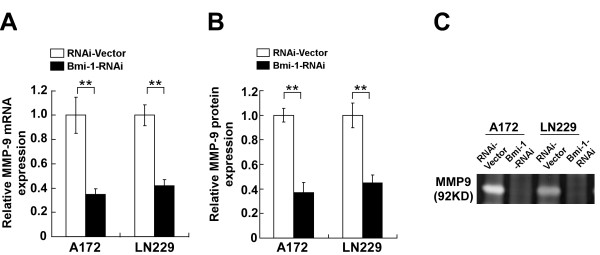
** Knockdown of Bmi-1 transcriptionally downregulates MMP-9 expression and activity. A**, Quantification MMP-9 mRNA expression levels in control cells and Bmi-1 RNAi-transfected cells; normalized to β-actin. **B**, ELISA quantification of MMP-9 protein in cell supernatants. **C**, Gelatin zymography assay of MMP-9 gelatinase activity in cell supernatants. RNAi-Vector: pSuper-retro-puro-vector. Error bars represent the mean ± SD of three independent experiments; ** *P* < 0.01

To construct an experimental model in which endogenous Bmi-1 expression was silenced, *Bmi-1* RNA interference (RNAi) sequences were cloned into the retroviral transfer vector pSuper-retro-puro, and retroviral production and infection were performed as previously described [18]. Western blotting confirmed that Bmi-1 protein expression was silenced in glioma cells transduced with pSuper-retro-puro-Bmi-1-RNAi retroviral vector (Figure 3A). Knocking down endogenous Bmi-1 dramatically reduced the migration and invasion of A172 and LN229 cells, and induced immotile and spheroid morphology (Figure 3B-E). Knockdown of Bmi-1 also significantly decreased the expression and activity of MMP-9 in glioma cells when compared with vector-control cells (Figure 4A-C).

### Bmi-1 induced expression of MMP-9 via activation of the NF-κB pathway

**Figure 5:**
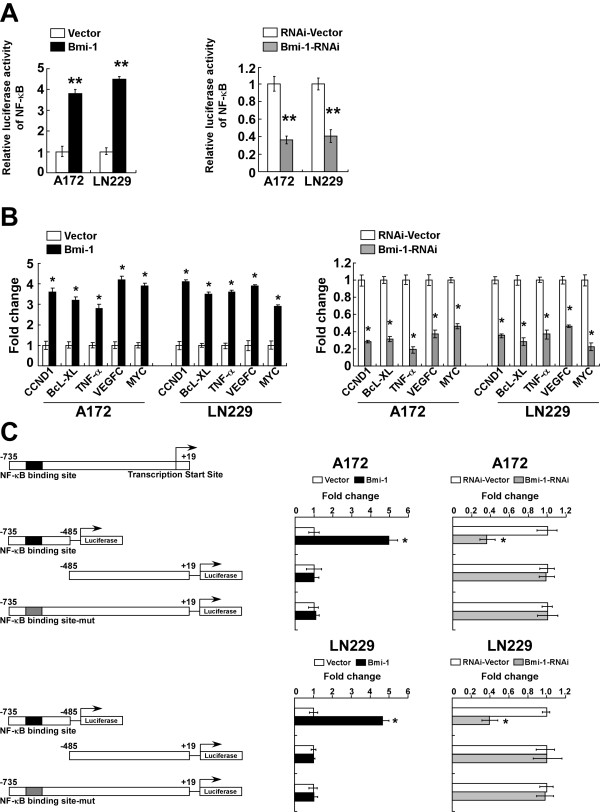
** Bmi-1 induces NF-kappaB transcriptional activity. A**, Luciferase reporter assay of NF-κB transcriptional activity in vector-control, Bmi-1 overexpressing (Bmi-1) and Bmi-1 silenced glioma cells (Bmi-1-RNAi). **B**, Real-time PCR analysis of NF-κB-regulated gene expression in vector-control, Bmi-1 overexpressing and Bmi-1 silenced glioma cells; GAPDH was used as the control gene. **C**, Left, schematic illustration of luciferase reporter gene construction using cloned fragments of the human *MMP-9 *promoter. Right, transactivation activity of luciferase reporter genes driven by *MMP-9 *promoter fragments (as indicated on the left) in vector-control, Bmi-1 overexpressing and Bmi-1 silenced glioma cells. Luciferase activity was normalized to *Renilla *luciferase activity. Vector: pMSCV-vector, RNAi-Vector: pSuper-retro-puro-vector. Error bars represent the mean ± SD of three independent experiments; * *P* < 0.05, ** *P* < 0.01.

We previously reported that Bmi-1 promoted NF-kappaB activation in glioma [18]. In the current study, we assessed the impact of Bmi-1 on NF-kappaB transcriptional activity in A172 and LN229 glioma cells using a luciferase reporter assay. As shown in Figure 5A, overexpression of Bmi-1 increased, whereas silencing of Bmi-1 inhibited the luciferase activity of the NF-kappaB reporter gene. As activation of NF-kappaB induces the transcription of a variety of NF-kappaB target genes, we performed semi-quantitative RT-PCR analysis to quantify the expression levels of selected NF-kappaB target genes, including *CCND1BcL-XLTNF-αVEGF-C* and *MYC*, in Bmi-1-overexpressing, -silenced and vector control-glioma cells. Compared to vector control-cells, overexpression of Bmi-1 upregulated the transcription of NF-kappaB target genes; whereas knockdown of Bmi-1 downregulated these target genes (Figure 5B).

Moreover, the luciferase activity of a reporter gene driven by the *MMP-9* promoter containing the NF-kappaB binding site increased significantly in Bmi-1-overexpressing cells and decreased in Bmi-1-silenced cells. Mutating the NF-kappaB binding site in the *MMP-9* promoter abrogated luciferase activity, and a *MMP-9* promoter fragment lacking the NF-kappaB binding site displayed no significant change in the luciferase activity in Bmi-1 overexpressing glioma cells (Figure 5C). Taken together, these results indicated that Bmi-1 promoted the transactivation activity of the NF-kappaB binding site present in the *MMP-9* promoter in glioma cells.

### Bmi-1 induced aggressiveness in glioma cells via the NF-kappaB/MMP-9 pathway

**Figure 6:**
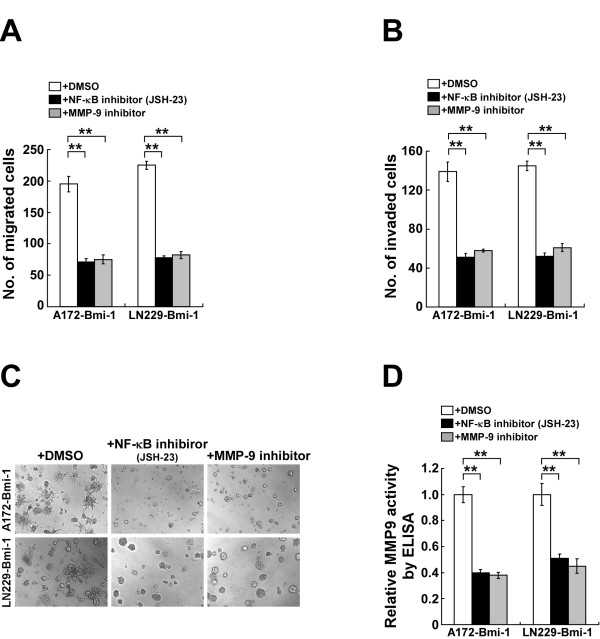
** Bmi-1 promotes an aggressive phenotype in glioma cells via activation of the NF-kappaB-MMP-9 pathway. **Bmi-1-overexpressing cells were treated with a NF-kappaB inhibitor JSH-23 at 30μM or MMP-9 inhibitor at 50μM. **A**, Quantification of cell migration in the Transwell assay (without matrigel). **B**, Quantification of cell invasion in the Transwell matrix penetration assay (with matrigel). **C**, Representative micrographs of the 3-D spheroid invasion assay. The experiments were repeated for at least three times with similar results. **D**, ELISA analysis of MMP-9 secretion. Error bars represent SD of three independent experiments; ** *P* < 0.01.

Next, we examined whether Bmi-1 increased the aggressiveness of glioma cells via activation of the NF-kappaB signaling pathway. The increased migratory and invasive ability of Bmi-1-overexpressing glioma cells were dramatically reversed by treatment with JSH-23, a specific NF-kappaB inhibitor that reduces the transcriptional activity of NF-kappaB, and these effects were accompanied by a reduction in MMP-9 expression (Figure 6A-D). These data suggested that functional activation of NF-kappaB was essential for the highly invasive phenotype induced in glioma cells by Bmi-1. Moreover, the invasivenes of Bmi-1-overexpressing glioma cells could be abrogated by an MMP-9 inhibitor, suggesting that MMP-9 played an important role in mediating Bmi-1-induced invasion of glioma cells (Figure 6A-D). Moreover, when we further examined the effect of Bmi-1 on the expression levels of MMP-2 and MMP-7 in A172 and LN229 glioma cells, our result showed that neither overexpressing nor silencing Bmi-1 had any an effect on the mRNA and protein levels of MMP-2 or MMP-7 (Additional file 2: Figure S1), implicating that MMP-9 might be a major mediator for Bmi-1 mediated invasiveness of glioma cells.

Collectively, these results indicated that the overexpression of Bmi-1 induced an aggressive phenotype in glioma cells by activating NF-kappaB signaling, leading to the upregulation of the NF-kappaB target gene MMP-9.

### Clinical relevance of Bmi-1 triggered NF-kappaB/MMP-9 activation in human gliomas

**Figure 7:**
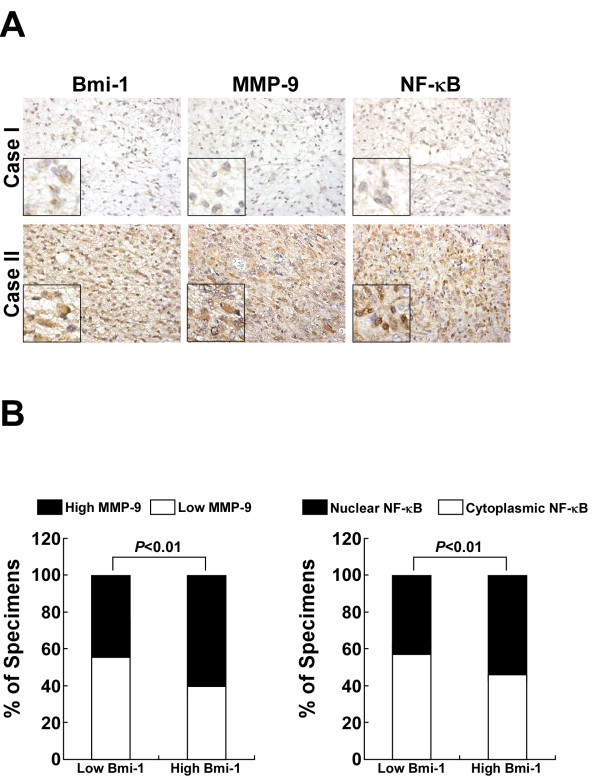
** Clinical relevance of Bmi-1 expression in human gliomas. A**, Bmi-1 levels in association with MMP-9 and NF-kappaB expression in 127 primary human glioma specimens. Two representative cases, diagnosed as WHO grade I (upper row) and grade III (lower row), are shown. The insets are enlarged images derived from the original pictures. **B**, Percentages of all specimens (upper) and specimens in grade IV (lower) showing low- or high-Bmi-1 expression relative to the levels of MMP-9 and nuclear or cytoplasmic NF-kappaB p65 localization, analyzed by IHC staining.

Lastly, we examined whether the NF-kappaB-activating effect of Bmi-1 in glioma cells found in our *in vitro* tests was clinically relevant. IHC analysis of 127 cases of clinical human glioma specimens revealed that upregulation of Bmi-1 was associated with in an upregulation of MMP-9 and nuclear localization of NF-kappaB (Figure 7A). Correlation studies in glioma specimens showed that Bmi-1 expression significantly correlated with the expression of MMP-9 (*P* < 0.01). Our results also exhibited that Bmi-1 expression and NF-kappaB nuclear localization were statistically correlated (Figure 7B, *P* < 0.01). Collectively, all these results further supported the notions that Bmi-1 induced MMP-9 expression, and that such effects involved activating NF-kappaB signaling.

## Discussion

The key finding of this study is that Bmi-1 may induce an aggressive phenotype in glioma via modulation of the NF-kappaB signaling. Previous studies have indicated that Bmi-1 is overexpressed and associated with poorer overall survival in glioma [18]. Our current study used glioma cell lines expressing intermediate levels of endogenous Bmi-1 as an experimental model, and by examining the effect of knocking down endogenous Bmi-1, or overexpressing ectopic Bmi-1, on the phenotype of glioma cells, we have identified that Bmi-1 activates NF-kappaB and subsequently upregulates MMP-9 expression, leading to increased migration and invasion of glioma cells.

As a member of the PcG family, Bmi-1 is overexpressed in various tumor types, including acute myeloid leukemia, lung cancer, ovarian cancer, nasopharyngeal carcinoma, breast cancer and colon cancer, suggesting that Bmi-1 represents a potential oncogene [16,35-39]. Furthermore, p16^INK4a^ and p14^ARF^ are targets of Bmi-1 suppression [40,41], and Bmi-1 has been found to promote cell proliferation by suppressing the p16/Rb and/or p14^ARF^/MDM2/p53 pathways [42,43]. Upregulation of Bmi-1 also induces the epithelial-mesenchymal transition (EMT), enhances the aggressiveness of human nasopharyngeal carcinoma cells and stabilizes Snail, a transcriptional repressor associated with EMT, via modulation of the PI3K/Akt/GSK-3β pathway [16]. Moreover, it has been reported that Bmi-1 can downregulate transcription of the tumor suppressor phosphatase and tensin homolog deleted on chromosome ten (PTEN) via a direct association with the *PTEN* gene locus [16]. Our current study indicates that Bmi-1 modulates the NF-kappaB/MMP-9 signaling pathway to mediate an aggressive phenotype in human glioma, suggesting that Bmi-1 may represent a potential therapeutic target for the treatment of glioma.

Activating mutations or amplification of oncogenes, such as epidermal growth factor receptor (EGFR) and phosphatidylinositol 3-kinase (PI-3 K), or loss of function in tumor suppressor genes, such as *p53* and *PTEN*, are involved in oncogenesis and the progression of glioma. The molecular mechanisms that mediate the aggressive phenotype in gliomas, however, are incompletely understood [44-46]. Furthermore, it is now well recognized that the low survival rate of glioma patients can be largely attributed to the highly invasive nature of glioma cells, which results in the destruction of surrounding brain tissue, and the invasiveness of glioma cells correlates with patient prognosis [2,3,44-47]. Most current therapies for the treatment of glioma are ineffective against invading cells. Characterization of the molecular mechanisms mediating invasion may provide a foundation for the development of new anti-glioma strategies. MMP-9, one member of the MMP family, is upregulated and associated with progression and poor prognosis in glioma [8]. Interestingly, numerous genes that promote the aggressiveness of glioma, including astrocyte elevated gene-1 (*AEG-1*), are also involved in the modulation of MMP-9 transcription [48]. Furthermore, multiple transcription factor-binding sites have been characterized in the upstream regulatory region of the *MMP-9* gene, including binding sites for the AP-1 and NF-kappaB transcription factors [8,49,50]. Moreover, AP-1 and NF-kappaB transcription factors can induce expression and activation of MMP-9 by interacting with these binding sites, and consequently promote tumor progression [48,51]. The present study demonstrates that Bmi-1 induces MMP-9 expression and activity via a mechanism associated with NF-kappaB activation, whereas blocking the activity of NF-kappaB drastically reduces the pro-invasive effect of Bmi-1 and preventes upregulation of MMP-9. Taken together, our data provide new insights in the development of novel strategies to prevent tumor invasion in glioma by inhibiting the expression of Bmi-1.

## Conclusions

In conclusion, this study demonstrates that Bmi-1 is upregulated and promotes an aggressive phenotype in glioma via activation of the NF-kappaB signaling pathway, leading to increased MMP-9 expression and activity. Bmi-1 may therefore represent a potential therapeutic target for improved treatment of human gliomas.

## Abbreviations

Bmi-1: B-cell-specific Moloney murine leukemia virus integration site 1; RNAi: RNA interference; MMP-9: Matrix metalloproteinase-9; PcG: Polycomb group; HOXA9: Homeobox A9; HOXC13: Homeobox C13; NF-kappaB: Nuclear factor kappaB; GBM: Glioblastoma multiform; FBS: Fetal bovine serum; DMSO: Dimethyl sulfoxide; ELISA: Enzyme-linked immunosorbent assay; shRNA: Short hairpin RNA; EMT: Epithelial-mesenchymal transition; EGFR: Epidermal growth factor receptor; PI-3 K: Phosphatidylinositol 3-kinase; AEG-1: Astrocyte elevated gene-1; VEGF-C: Endothelial growth factor-C; hr: Hour; mg: Microgram; μl: Microliter; μM: Micromolar; ml: Milliter; PCR: Polymerase chain reaction; PTEN: Phosphatase and tensin homolog deleted on chromosome ten.

## Competing interests

The authors declare that they have no competing interests.

## Authors’ contributions

LJ and JW performed most of the laboratory analyses, wrote the preliminary manuscript and were actively involved in the field work. LS developed the improved all laboratory protocols and performed all laboratory analyses assays. LS and LL prepared cell culture assays for biological function analyses. LJ and JW analyzed genes and proteins expression and analyses. All statistical analyses were done under the supervision of LJ together with JW and LS. JL and ML designed and supervised the study, were involved in data analyses and wrote the finalized manuscript. All authors read and approved the final manuscript.

## Pre-publication history

The pre-publication history for this paper can be accessed here:

http://www.biomedcentral.com/1471-2407/12/406/prepub

## Supplementary Material

Additional file 1 **Table S1.** Clinicopathological characteristics of studied patients in gliomas [8]. Click here for file

Additional file 2 ** Figure S1. **The expression level of MMP-2 and MMP-7. **A**, Real-time PCR quantification of *MMP-2 and MMP-7 *mRNA expression levels in Bmi-1-overexpressing and Bmi-1-silencing cells. *MMP-2 and MMP-7 *expression levels are presented as fold changes relative to vector-control cells and normalized to GAPDH. The primiers, *MMP-2-up*: CAGGGAATGAGTACTGGGTCTATT; *MMP-2-dn*: ACTCCAGTTAAAGGCAGCATCTAC; *MMP-7-up*: AGCCAAACTCAAGGAGATGC; *MMP-7-dn*: ACTCCACATCTGGGCTTCTG. **B**, ELISA assay of secreted MMP-2 and MMP-7 protein activity in cell supernatants. Error bars represent the mean ± SD of three independent experiments. Click here for file
