# Supplementary material for: Bmi-1 promotes the aggressiveness of glioma via activating the NF-kappaB/MMP-9 signaling pathway
Source: BMC Cancer. 2012 Sep 11;12:406. doi: 10.1186/1471-2407-12-406 (PMC3502583; doi:10.1186/1471-2407-12-406)
Supplement: Additional file 1 — Table S1. Clinicopathological characteristics of studied patients in gliomas [8]. [file 1471-2407-12-406-S1.doc]

**Supplemental Table 1**.

Clinicopathological characteristics of studied patients in gliomas [8]

| **Factor** | **No.** | **(%)** |
| --- | --- | --- |
| **Gender** |  |  |
| Male | 92 | 72.4 |
| Female | 35 | 27.6 |
| **Age (years)** |  |  |
| ≤ 45 | 85 | 66.9 |
| > 45 | 42 | 33.1 |
| **Glioma histopathology (WHO grading)** |  |  |
| Grade I | 12 | 9.4 |
| Grade II | 33 | 26.0 |
| Grade III | 59 | 46.5 |
| Grade IV | 23 | 18.1 |
| **Patient survival (n=127)** |  |  |
| Alive | 41 | 32.3 |
| Deceased | 86 | 67.7 |
