# Supplementary figures and images for: Bmi-1 promotes the aggressiveness of glioma via activating the NF-kappaB/MMP-9 signaling pathway
Source: BMC Cancer. 2012 Sep 11;12:406. doi: 10.1186/1471-2407-12-406 (PMC3502583; doi:10.1186/1471-2407-12-406)

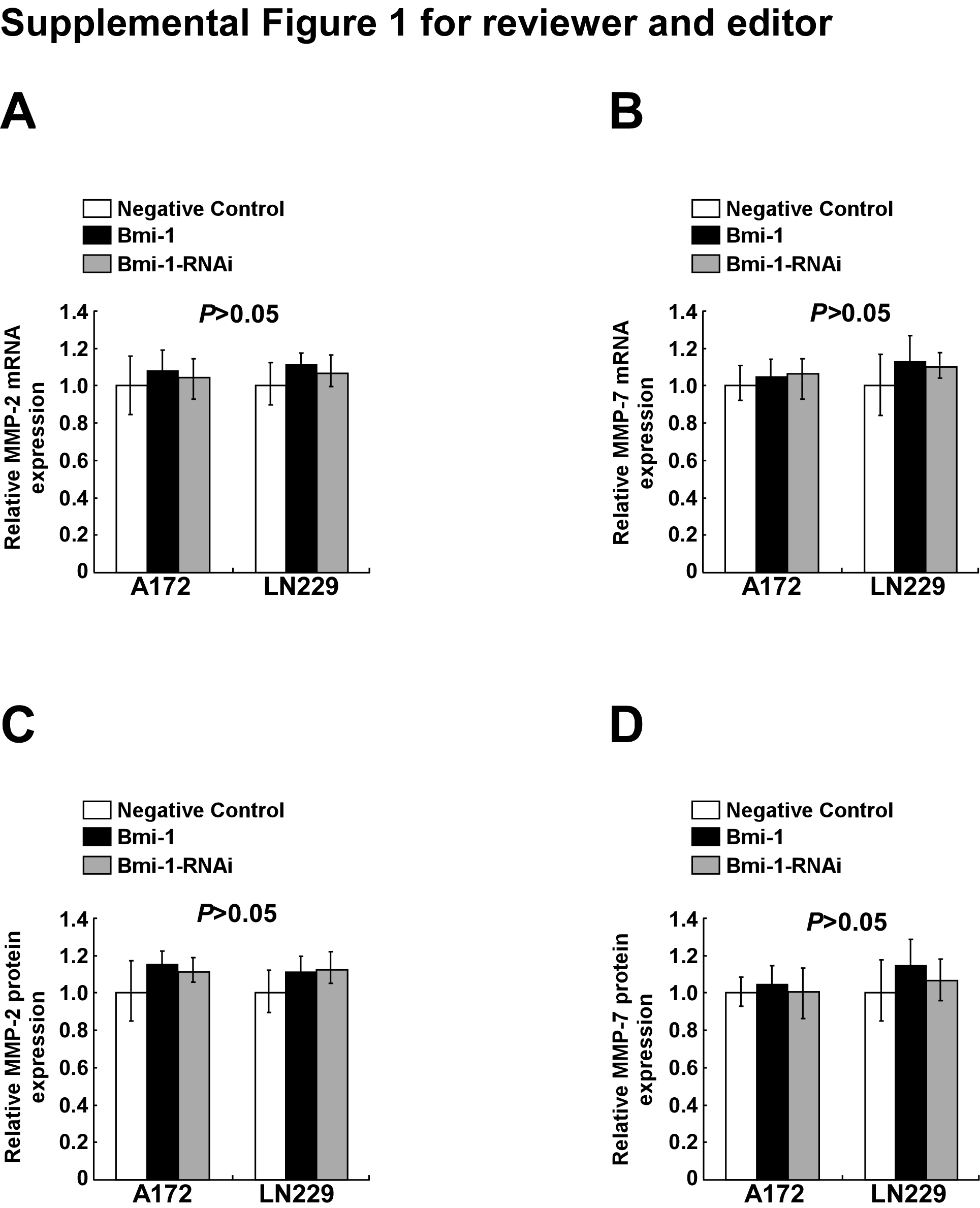

Supplement: Additional file 2 — Figure S1. The expression level of MMP-2 and MMP-7. A, Real-time PCR quantification of MMP-2 and MMP-7 mRNA expression levels in Bmi-1-overexpressing and Bmi-1-silencing cells. MMP-2 and MMP-7 expression levels are presented as fold changes relative to vector-control cells and normalized to GAPDH. The primiers, MMP-2-up: CAGGGAATGAGTACTGGGTCTATT; MMP-2-dn: ACTCCAGTTAAAGGCAGCATCTAC; MMP-7-up: AGCCAAACTCAAGGAGATGC; MMP-7-dn: ACTCCACATCTGGGCTTCTG. B, ELISA assay of secreted MMP-2 and MMP-7 protein activity in cell supernatants. Error bars represent the mean ± SD of three independent experiments. [file 1471-2407-12-406-S2.tiff]
